# The effect of obesity on intraoperative complication rates with hysteroscopic compared to laparoscopic sterilization: a retrospective cohort study

**DOI:** 10.1186/s40834-016-0008-3

**Published:** 2016-02-23

**Authors:** Rachel Shepherd, Christina A. Raker, Gina M. Savella, Nan Du, Kristen A. Matteson, Rebecca H. Allen

**Affiliations:** Department of Obstetrics & Gynecology, Warren Alpert Medical School of Brown University, Women & Infants Hospital, 101 Dudley St, Providence, RI 02905 USA

**Keywords:** Tubal sterilization, Hysteroscopy, Laparoscopy, Essure, Obesity, Complications

## Abstract

**Background:**

Surgical sterilization is a common method of contraception. There have been few studies evaluating the effect of obesity on procedural complications with either laparoscopic or hysteroscopic methods of sterilization. The purpose of this study was to compare the incidence of intraoperative complications of hysteroscopic tubal occlusion with laparoscopic tubal ligation among obese and nonobese women.

**Methods:**

This retrospective cohort study compared women undergoing interval laparoscopic or hysteroscopic sterilization in the operating room between September 2009 and December 2011 at a single hospital. Serious complications included: unintended surgery, uterine perforation, anaphylaxis, blood transfusion, infection requiring antibiotics, hospital admission, fluid overload, myocardial infarction, and venous thromboembolism. Post-operative events included: nausea/vomiting, doctor evaluation or additional pain medication required in the recovery room, and emergency department visit within 2 weeks of surgery. The association between sterilization type and incidence of complications was examined overall, separately by BMI group, and also among patients who received general anesthesia.

**Results:**

A total of 433 laparoscopic and 277 hysteroscopic procedures were reviewed. The BMI distribution of the sample was 35 % normal weight, 31 % overweight, and 34 % obese which is comparable to the general US female population. No life-threatening events were identified. Serious complications were similar with 20 (4.6 %) in the laparoscopic group and 11 (4.0 %) in the hysteroscopic group (*p* = 0.9). The most common serious complications were bleeding from the tube, cervical laceration, and uterine perforation. Although not statistically significant, women with a BMI of 30 or greater had only 1 (1 %) serious complication in the hysteroscopic group compared to 7 (5.2 %) in the laparoscopic group. Postoperative events were increased in the laparoscopic group (16.2 %) compared to the hysteroscopic group (6.9 %), especially among overweight and obese women (*p* <0.01). Failure to complete the intended bilateral occlusion occurred for 14 women in the hysteroscopic group compared to just one woman in the laparoscopic group (*p* <0.001).

**Conclusion:**

Both laparoscopic and hysteroscopic tubal sterilization are safe with few serious complications based on these data. No cases of laparotomy, blood transfusion, or life-threatening events were identified. There was no difference in serious complication rate by sterilization method. Overweight and obese women were no more likely to experience a serious complication with either method than women with a BMI <25. There were fewer postoperative events (*p* <0.01) with hysteroscopic sterilization, but far fewer failed laparoscopic procedures (*p* <0.001). These study findings can be used to enhance sterilization counseling.

## Background

Surgical sterilization is one of the most commonly used methods of contraception for women in the United States with over 600,000 procedures performed each year [1]. The landmark United States Collaborative Review of Sterilization (CREST) study analyzed complication rates among 9,475 women undergoing interval laparoscopic tubal ligation (LTL) from 1978 to 1987. Complications, defined as performance of an unintended major surgery at the time of sterilization, occurred in 0.9 per 100 women [2]. This strict definition of complications did not include post-operative events that a patient might factor into her decision to proceed with an elective sterilization. Unlike LTL, hysteroscopic sterilization (HS) does not require an abdominal incision or peritoneal cavity distention. A British study which defined complications broadly including vasovagal reactions, cervical tear and bleeding, tubal perforation, uterine perforation, postoperative pain, and nausea/vomiting found that outpatient HS was associated with fewer complications than operating room LTL (11 % vs. 27 %) [3].

The relationship between obesity and complication rates with different types of surgical sterilization has not been completely elucidated. With the high prevalence of obesity in the US, health care providers need data to assess the risks associated with surgical contraception in obese women [4]. Overweight and obese women are more likely to opt for tubal sterilization compared to normal weight women [5, 6]. Providers often counsel obese women that laparoscopic sterilization may be associated with a higher risk of complications compared to hysteroscopic sterilization. Indeed, the CREST study concluded that obese women have a slightly higher risk of complications with laparoscopic tubal ligation compared to nonobese women (OR 1.7; 95 % CI 1.2, 2.6) [2]. However, there is a paucity of objective data to verify that that HS is a safer option for obese women. The goals of this study were to compare the risk of complications between HS and LTL and to stratify complication rates by BMI. We hypothesized that complication rates would be higher with laparoscopic tubal sterilization compared to hysteroscopic, and that obese women undergoing surgical sterilization would have a higher risk of complications compared to non-obese women.

## Methods

This retrospective cohort study compared all women who underwent interval LTL and HS at a large academic women’s hospital between September 2009 and December 2011. Cases were identified by a search of the hospital’s electronic medical record by surgical procedure. At the time of the study, all laparoscopic procedures and the majority of hysteroscopic sterilization procedures in the community were performed in the hospital’s operating room. The choice of sterilization procedure was at the discretion of the surgeon. The type of laparoscopic occlusion was identified from the operative report. Data were collected from medical records and included information on demographics, medical and surgical history from the admission note, surgical procedure, and any intraoperative or postoperative complications. Overall surgical and recovery room times were collected from the medical record as documented by nursing staff. Any emergency department visit within 2 weeks of surgery was recorded and the physician notes were reviewed to determine if the visit was related to the surgery. This study was approved by the Women & Infants Hospital Institutional Review Board.

The main independent variables were body mass index (BMI) and type of sterilization (LTL vs. HS). BMI was categorized according to standard criteria: less than 25 kg/mg^2^, normal weight; 25 to 29.9 kg/m^2^, overweight; and 30 or more kg/m^2^, obese [7]. Height and weight were routinely measured on the day of surgery by the preoperative staff. The dependent variables were serious complications and post-operative events. Serious complications included: unintended surgery (laparotomy, major blood vessel repair, resection of tube, bleeding from tube, oophorectomy, or repair of the bowel, bladder, cervix, or uterus), life-threatening event (anaphylaxis, myocardial infarction, and venous thromboembolism), uterine perforation, blood transfusion, infection requiring antibiotics, hospital admission, and fluid overload leading to pulmonary edema. Post-operative events included: nausea/vomiting, doctor evaluation in the post-anesthesia care unit (PACU), pain requiring additional medication in the PACU (defined as more than the standard single dose of postoperative pain medication), and an emergency department visit within 2 weeks of surgery. Patients with multiple serious complications or post-operative events were counted only once in each applicable category of complications for data analysis. We also collected information on whether the procedure was able to be performed as intended or whether conversion to laparoscopy or laparotomy was required.

The sample size calculation focused on detecting a higher incidence of complications (serious and/or postoperative) among laparoscopic sterilization patients, compared to hysteroscopic sterilization patients. The null hypothesis was no difference in the incidence of complications between the two procedures. Based on a literature review and institution-specific data, we assumed that the incidence of having any complication was 7 and 2 % for the laparoscopic and hysteroscopic groups, respectively. After an initial review of medical records, we estimated that there were approximately twice as many laparoscopic sterilizations as hysteroscopic sterilizations. Therefore, we used a ratio of 2:1 for the comparison groups in our calculation, as opposed to assuming equally-sized groups. We estimated that 474 laparoscopic and 237 hysteroscopic patients were required to detect a 5 % absolute difference (7 % vs. 2 %) in the incidence of any complication with a two-sided alpha of 0.05 and 80 % power.

Statistical analyses were performed using STATA 10 software (StataCorp, College Station, TX). Categorical variables were analyzed by chi-square and Fisher’s exact tests and continuous variables were analyzed by T test and Wilcoxon rank-sum test. The association between sterilization type and incidence of complications was examined overall, separately by BMI group, and also among patients who received general anesthesia. All *p*-values are two-tailed with *p* <0.05 considered statistically significant.

## Results

During the study period, 710 women underwent interval surgical sterilization, 433 via the laparoscopic approach and 277 via the hysteroscopic approach. Of the laparoscopic procedures, 407 (94 %) were performed with Falope rings, the remainder with bipolar tubal coagulation. The hysteroscopic sterilization method utilized in all cases was Essure (Bayer Healthcare Pharmaceuticals, Whippany, NJ). The mean age was 33.7 years (±6.3) and there was no significant difference in gravidity, parity, history of abdominal surgery or medical comorbidities between the two groups (Table 1). More women in the hysteroscopic sterilization group had Medicaid insurance (55.1 %) compared to the laparoscopic group (46.3 %). A total of 234 (33.6 %) women were obese. The hysteroscopic sterilization group had a slightly higher mean BMI of 29.6 kg/m^2^ compared to 27.9 kg/m^2^ for the laparoscopic group (*p* = 0.002) (Table 1). All of the laparoscopic tubal ligations and the majority of the hysteroscopic sterilizations (72 %) were performed under general anesthesia. Both surgical time and recovery time were greater for the LTL group than the hysteroscopic group (Table 2). Estimated blood loss was low for both procedures (<25 mL).

**Table 1:** Clinical characteristics of study sample by method of sterilization (*N* = 710)

	Total (*n* = 710)	Laparoscopic (*n* = 433)	Hysteroscopic (*n* = 277)	*P* value
Age				
Mean (SD)	33.7 (6.3)	33.6 (6.2)	33.9 (6.5)	0.6
Race/ethnicity				
Hispanic/Latina	201 (30.0)	121 (29.7)	80 (30.4)	0.5
White	362 (54.0)	224 (54.9)	138 (52.5)	
Black	47 (7.0)	23 (5.6)	24 (9.1)	
Asian	13 (1.9)	9 (2.2)	4 (1.5)	
Other	48 (7.2)	31 (7.6)	17 (6.5)	
Gravidity				
Median (Range)	3 (0–11)	3 (0–11)	3 (0–11)	0.7
Parity				
Median (Range)	2 (0–8)	2 (0–8)	2 (0–6)	0.2
Insurance				
Medicaid	315 (49.7)	179 (46.3)	136 (55.1)	0.09
Private	298 (47.0)	195 (50.4)	103 (41.7)	
Self-pay	21 (3.3)	13 (3.4)	8 (3.2)	
BMI				
Mean (SD)	28.5 (6.8)	27.9 (6.1)	29.6 (7.6)	0.002
BMI class				
<25	247 (35.5)	160 (37.5)	87 (32.3)	0.04
25–29.9	215 (30.9)	133 (31.2)	82 (30.5)	
30–34.9	124 (17.8)	79 (18.5)	45 (16.7)	
35–39.9	68 (9.8)	38 (8.9)	30 (11.2)	
≥40	42 (6.0)	17 (4.0)	25 (9.3)	
Any comorbidities				
Yes	326 (45.9)	193 (44.6)	133 (48.0)	0.4
No	384 (54.1)	240 (55.4)	144 (52.0)	
Any abdominal surgery				
Yes	288 (40.6)	179 (41.3)	109 (39.4)	0.6
No	422 (59.4)	254 (58.7)	168 (60.7)	
Abdominal surgeries				
Cesarean section	206 (29.0)	127 (29.3)	79 (28.5)	0.9
Appendectomy	43 (6.1)	24 (5.5)	19 (6.9)	0.5
Cholecystectomy	66 (9.3)	40 (9.2)	26 (9.4)	1.0
Umbilical hernia repair	12 (1.7)	4 (0.9)	8 (2.9)	0.07
Gastric bypass	13 (1.8)	6 (1.4)	7 (2.5)	0.4
Other laparoscopy/laparotomy	59 (8.3)	39 (9.0)	20 (7.2)	0.5

Mean (SD) or number (%). Numbers may not sum to total due to missing data: race/ethnicity (*n* = 39, 5.5 %), number of cesarean sections (*n* = 39, 5.5 %), gravidity (*n* = 18, 2.5 %), parity (*n* = 10, 1.4 %), insurance (*n* = 76, 10.7 %), and body mass index -BMI (*n* = 14, 2.0 %)

**Table 2:** Surgical characteristics of sample by method of sterilization

	Total	Laparoscopic	Hysteroscopic	*p*-value
Type of anesthesia				
General	630 (89.2)	433 (100)	197 (72.2)	<0.0001
Local only	8 (1.1)	0	8 (2.9)	
Spinal	9 (1.3)	0	9 (3.3)	
Intravenous sedation	59 (8.4)	0	59 (21.6)	
Surgical time (minutes)				
Median (IQR)	23 (15–34)	29 (21–40)	15 (11–22)	<0.0001
Recovery time (minutes)				
Median (IQR)	70 (58–89)	72 (60–90)	66 (55–86)	0.005
Estimated blood loss (cc)				
Median (IQR)	10 (5–20)	10 (5–20)	5 (5–10)	<0.0001

Number (%). Numbers may not sum to total due to missing data: anesthesia type (*n* = 4, 0.6 %), and estimated blood loss (*n* = 17, 2.4 %) *IQR* = interquartile range

The overall incidence of serious complications was 4.4 % for both laparoscopic and hysteroscopic sterilization. Overall, the most common serious complication in the LTL group was bleeding from the tube with a total of ten cases (Table 3). In contrast, the most common serious complication in the hysteroscopic group was uterine perforation with four cases. No cases of laparotomy, blood transfusion, infection requiring antibiotics, or life-threatening events were identified with either sterilization approach. No statistically significant differences were noted in serious complication rates by weight category between sterilization methods (Table 4). The one group that had a lower risk was obese women receiving HS. No clear trends emerged to suggest an underpowered negative result. This did not change when the data were controlled for prior abdominal surgery (data not shown). The seven serious complications among the obese women in the LTL group included two cervical repairs, two episodes of bleeding from the tube, one bleeding from tube and resection of tube, one uterine perforation, and one hospital admission. The one serious complication among obese women in the hysteroscopic group was fluid overload.

**Table 3:** Type of *serious* complications^b^ among all patients (*N* = 710)

Serious complication, number (%)^a^	Laparoscopic (*n* = 433)	Hysteroscopic (*n* = 277)	*P* value
Resection of tube	2 (0.5)	1 (0.4)	1.0
Bleeding from tube	10 (2.3)	2 (0.7)	0.1
Cervical repair	4 (0.9)	3 (1.1)	1.0
Uterine perforation	2 (0.5)	4 (1.4)	0.2
Fluid overload	0	1 (0.4)	0.4
Hospital admission	4 (0.9)	0 (0)	0.2

^a^There were no cases of laparotomy, repair major blood vessel, oophorectomy, bladder repair, uterine repair, bowel repair, anaphylaxis, infection requiring antibiotics, transfusion, myocardial infarction, or venous thromboembolism

^b^For each complication listed, the values presented are the number of patients with the complication and the proportion out of all patients in each group The p-values were calculated separately for each complication

**Table 4:** Incidence of *serious* complication by BMI and method of sterilization^b^ (*N* = 710)

Serious complication^a^	Total	Laparoscopic (*n* = 433)	Hysteroscopic (*n* = 277)	OR (95 % CI)	*P* value
Overall	31 (4.4)	20 (4.6)	11 (4.0)	0.9 (0.4-1.9)	0.9
BMI <25	12 (4.9)	7 (4.4)	5 (5.8)	1.3 (0.3-5.0)	0.8
BMI 25–25.9	10 (4.7)	5 (3.8)	5 (6.1)	1.7 (0.4-7.5)	0.5
BMI ≥30	8 (3.4)	7 (5.2)	1 (1.0)	0.2 (0.004-1.5)	0.1

Number (%). *BMI* body mass index

^a^Patients with more than one serious complication are counted just once

^b^Numbers may not sum to total due to missing BMI data (*n* = 14, 2.0 %)

Failure to perform the intended procedure occurred for 14 women (5 %) in the hysteroscopic group compared to just one woman (0.2 %) in the laparoscopic group (*p* <0.001). Of these 14 cases, there were seven among normal weight women, six among overweight women, and 1 in an obese woman. Eight were caused by an inability to pass the microinsert catheter into the tubal ostia or failure to deploy the microinsert, three were due to inadequate visualization of the tubal ostia, and three were attributable to suspected uterine perforations. Nine of these cases were converted to laparoscopic tubal ligations and one woman had a copper intrauterine device (IUD) placed. For the hysteroscopic sterilization group, follow-up hysterosalpingogram (HSG) data were available for 178 women (64 %). Among these women, 167 (94 %) HSGs demonstrated bilateral tubal occlusion on the three-month HSG. The remainder of the women are either presumed to not have followed-up for the HSG or obtained the test at an outside institution. The outcome of repeat 6-month HSGs is not known. In the laparoscopy group, one woman was not sterilized because dense pelvic adhesions obscured the tubes. A laparotomy was not performed.

The overall incidence of postoperative events was 16.2 % for laparoscopic and 6.9 % for hysteroscopic sterilization. More women in the laparoscopic group required additional medications for pain in the PACU and more visited the emergency department visit within 2 weeks after surgery for abdominal pain (71 %) related to the surgery, followed by wound complaints (12.5 %) (Table 5). The other four visits were for chest pain, urinary retention, dizziness, and constipation. There were no hospitalizations as a result of these visits. When stratified by BMI and receipt of general anesthesia, there was no significant difference between the laparoscopic approach and the hysteroscopic approach in incidence of postoperative events for normal weight and overweight women (Table 6). Among obese women, there was a statistically significant increase in incidence of postoperative events with the laparoscopic approach (17.2 %) when compared to 6 % with the hysteroscopic approach (*p*=<0.05).

**Table 5:** Type of *postoperative* events among patients (N = 710)

Event^a^	Laparoscopic (*n* = 433)	Hysteroscopic (*n* = 277)	*P* value
Nausea/vomiting	6 (1.4)	3 (1.1)	1.0
Doctor evaluation in PACU	20 (4.6)	10 (3.6)	0.6
Pain requiring add’l meds	37 (8.6)	9 (3.3)	0.005
Emergency department visit within 2 weeks	21 (4.9)	3 (1.1)	0.005

Number (%). *PACU* post-anesthesia care unit ^a^Patients with more than one complication are counted in each applicable complication

**Table 6:** Incidence of *postoperative* event by BMI and method of sterilization (*N* = 710)

				*All patients*		*General anesthesia* ^*b*^	
Postoperative event^a^	Total	Laparoscopic (*n* = 433)	Hysteroscopic (*n* = 277)	OR (95 % CI)	*P* value	OR (95 % CI)	*P* value
Overall	89 (12.5)	70 (16.2)	19 (6.9)	0.4 (0.2–0.7)	0.0002	0.5 (0.2–0.8)	0.006
BMI <25	27 (10.9)	19 (11.9)	8 (9.2)	0.8 (0.3–1.9)	0.7	1.0 (0.3–2.7)	1.0
BMI 25–25.9	31 (14.4)	26 (19.6)	5 (6.1)	0.3 (0.08–0.8)	0.008	0.4 (0.1–1.1)	0.06
BMI ≥30	29 (12.4)	23 (17.2)	6 (6.0)	0.3 (0.1–0.8)	0.02	0.3 (0.07–0.9)	0.02

Number (%). *BMI* body mass index

^a^Patients with more than one postoperative event are counted just once

^b^Included 433 laparoscopic tubal ligation patients and 197 hysteroscopic sterilization patients who received general anesthesia

## Discussion

In this retrospective cohort study, there was a greater incidence of postoperative events among obese women who had a laparoscopic sterilization compared to obese women who had hysteroscopic sterilization after controlling for receipt of general anesthesia. No life-threatening complications were noted. Serious complications were too infrequent to discern a difference among BMI categories when comparing sterilization methods. The serious complication rate ranged from 3.8 to 6.1 % for all categories except in obese women sterilized by hysteroscopy who only had a 1 % complication rate. No statistically significant difference was noted. More women in the hysteroscopic group had failed procedures compared to the laparoscopic group, unrelated to obesity. The rate of failure of microinsert placement in this study (5 %) is no different than that reported in the literature for these procedures [8]. Similar to another study, in this investigation BMI did not influence success of bilateral microinsert placement [9].

Although previous studies on female sterilization have compared the risk of complications for LTL and HS methods in a general population and the risk of complications for obese women undergoing laparoscopic sterilization, this is the first research on the safety of hysteroscopic sterilization for obese women. Hysteroscopic sterilization was safe for obese women with the most common serious complication being uterine perforation (1.4 %). The incidence of other serious complications was extremely low.

While surgical sterilization overall is very safe, the availability of equally effective long-acting reversible contraceptives (IUDs and implants) is important to consider. Women desire to understand the risk of surgery and how their BMI may alter their risk of a surgical complication or a complicated post-operative recovery. The American College of Obstetricians and Gynecologists recommends minimally invasive approaches for surgery in obese women if possible [10]. Laparoscopy in the morbidly obese may require special surgical techniques in terms of instruments and trocar placement [11]. In terms of approach, the hysteroscopic method is less invasive than the laparoscopic method of sterilization and may be preferred in obese women provided they are willing to comply with the 3-month hysterosalpingogram requirement and understand the success rate of bilateral tubal occlusion with the microinserts. All women, including obese women, should be offered long-acting reversible contraceptives (LARC) as an alternative to sterilization. In addition, vasectomy for the male partner should be discussed as it would obviate risks for the obese woman entirely.

The strengths of this study include a large, diverse patient population undergoing surgery with multiple academic faculty and community-based providers. There is no one standard way to define serious or major complications for elective surgery. Since tubal sterilization is considered an elective procedure and safer options with equal efficacy are available (LARC), a broader definition of complications was selected that included postoperative adverse events in addition to the usual intraoperative events. These minor events may be significant to a patient choosing between surgery or less invasive contraceptive methods or in selecting the specific type of sterilization. A weakness of the study is that complications were not classified via a validated system.

A limitation to this study was the exclusion of women who had outpatient procedures. At the time the study was performed, the majority of hysteroscopic and laparoscopic sterilizations in the community were performed in the hospital operating room. Nevertheless, previous research has found no significant difference in complication rates between HS performed in-office and those performed in a hospital operating room [8, 9]. Furthermore, controlling for the receipt of general anesthesia did not alter the results. Data collection was confined to available surgical and medical records and therefore did not account for long-term complications like failure of the sterilization (pregnancy) or patient-reported outcomes such as satisfaction and quality of life. In addition, the choice of sterilization procedure was at the discretion of the surgeon and likely based on multiple factors including patient BMI, history of abdominal surgery and medical problems, and preference of the surgeon. As a retrospective study, potential bias includes the selection of the procedure. Women with a BMI ≥40 were over twice as likely to receive hysteroscopic sterilization. In addition, data on surgeon experience was not collected for this study. How surgeon experience may have affected the results is uncertain. Finally, given that the vast majority of laparoscopic procedures were performed with the Falope Ring, whether other modalities would have been associated with less postoperative pain is unknown.

Studying the experience of women with varied BMI who select surgical sterilization is important because studies who that obese women are more likely to choose surgical sterilization [5, 12]. These data define the incidence of complications and the postoperative experience from laparoscopic compared to hysteroscopic sterilization by BMI. While the data are from a single institution, the demographic of the population is similar to many populations in the US and will improve the provider’s ability to thoughtfully tailor counseling, based on their BMI, about risks and adverse experiences to patients considering elective surgical sterilization.

## Conclusions

Both laparoscopic and hysteroscopic tubal sterilization are safe with few serious complications based on these data. No cases of laparotomy, blood transfusion, or life-threatening events were identified. There was no difference in serious complication rate by sterilization method. Overweight and obese women were no more likely to experience a serious complication with either method than women with a BMI <25. There were fewer postoperative events (p <0.01) with hysteroscopic sterilization, but far fewer failed laparoscopic procedures (p <0.001). These study findings can be used to enhance sterilization counseling.
